# Optimizing bacteriophage treatment of resistant *Pseudomonas*

**DOI:** 10.1128/msphere.00707-23

**Published:** 2024-06-27

**Authors:** Laura Ulrich, Leon X. Steiner, Christoph Giez, Tim Lachnit

**Affiliations:** 1 Zoological Institute, Christian-Albrechts Universität zu Kiel, Kiel, Germany; 2 RD3 Marine Ecology, RU Marine Symbioses, GEOMAR Helmholtz Centre for Ocean Research, Kiel, Germany; University of Michigan, Ann Arbor, Michigan, USA

**Keywords:** bacteriophages, phage therapy, antimicrobial resistance, *Pseudomonas*

## Abstract

Phage therapy is increasing in relevance as an alternative treatment to combat antibiotic resistant bacteria. Phage cocktails are the state-of-the-art method of administering phages in clinical settings, preferred over monophage treatment because of their ability to eliminate multiple bacterial strains and reduce resistance formation. In our study, we compare monophage applications and phage cocktails to our chosen method of phage sequential treatments. To do so, we isolated four novel bacteriophages capable of infecting *Pseudomonas alcaligenes* T3, a close relative of *P. aeruginosa*, and characterized them using sequencing and transmission electron microscopy. While investigating monophage treatments, we observed that different phage concentrations had a strong impact on the timing and amount of resistance formation. When using phage cocktails, we observed that *P. alcaligenes* were capable of forming resistance in the same timespan it took them to become resistant to single phages. We isolated mutants resistant to each single phage as well as mutants exposed to phage cocktails, resulting in bacteria resistant to all four phages at once. Sequencing these mutants showed that different treatments yielded unique single nucleotide polymorphism mutation patterns. In order to combat resistance formation, we added phages one by one in intervals of 24 h, thus managing to delay resistance development and keeping bacterial growth significantly lower compared to phage cocktails.

**IMPORTANCE:**

WHO declared antimicrobial resistance a top threat to global health; while antibiotics have stood at the forefront in the fight against bacterial infection, the increasing number of multidrug-resistant bacteria highlights a need to branch out in order to address the threat of antimicrobial resistance. Bacteriophages, viruses solely infecting bacteria, could present a solution due to their abundance, versatility, and adaptability. For this study, we isolated new phages infecting a fast-mutating *Pseudomonas alcaligenes* strain capable of forming resistance within 30 h. By using a sequential treatment approach of adding one phage after another, we were able to curb bacterial growth significantly more compared to state-of-the-art phage cocktails.

## INTRODUCTION

The rising threat of multidrug-resistant bacteria is a well-known, global concern (1, 2). In response, modern research has come together to illuminate the problem from multiple angles. The medical sector, where antimicrobial resistance is limiting treatment options and in turn increasing death rates, especially due to *Pseudomonas aeruginosa*, *Staphylococcus aureus*, and other “ESKAPE” organisms (3), has been at the forefront of this endeavor. Other fields followed suit, investigating the impact of multidrug-resistant bacteria on the human microbiota (4), horizontal transmission of resistance genes (5), and the accelerating effect of overabundant use of antibiotics in human and veterinary medicine on propagating antimicrobial resistance (6).

Antimicrobial stewardship programs limiting the use of antibiotics and increasing preventive measures have been shown to shorten hospital stays and reduce treatment costs (7, 8) but were challenged by the recent COVID pandemic which resulted in an increase in antibiotic use (9). Ultimately, while preventive measures are important, antimicrobial resistance is already widespread, calling forth a need for remedial action. One such endeavor is the search for new antibiotics (10, 11) which brings its own challenges, since antibiotics need to target a limited set of cellular processes to avoid cytotoxicity (12) and their development is costly (13). One potential solution to this crisis is bacteriophages.

Phages are viruses capable of specifically and selectively killing bacteria without causing adverse effects in eukaryotes (14, 15). Their earliest medical application was performed in 1919 by Félix Hubert d’Hérelle, who used phages to cure chicken infected with *Salmonella gallinarum* (16). In the following decade, phages were used as antimicrobials to combat the likes of cholera and the bubonic plague (17), until they were overshadowed by antibiotics, which were easier to store and manufacture (18). To this day, bacteriophages are almost exclusively used for medical purposes in Eastern European countries (19, 20), though they are now experiencing a surge globally (21). When used in clinical settings, bacteriophages are often applied in the form of phage cocktails, which consist of a multitude of different bacteriophages merged into a single therapeutic solution. Examples of this are phage cocktails prepared against *M. tuberculosis* (22), *E. coli* (23), and multiple other organisms, with the overall consensus that cocktails are preferred over monophage treatments (24, 25).

An application of phage treatments other than cocktails involves the sequential administration of one phage after another in sequence. Such experiments have been performed on *P. aeruginosa* in wax moths, where it was found that sequential treatment using four phages was equally as effective as phage cocktail treatments, at least considering short-term outcomes (26). When pairs consisting of two phages were used against *P. aeruginosa*, it was found that the sequence in which phages were administered, had a strong impact on the treatment’s efficiency (27).

Our goal is to build on previous works utilizing phage sequential treatment (PST) and further optimize phage treatments against bacteria prone to high mutation rates. For this purpose, we tested our treatment approach on *Pseudomonas alcaligenes* T3, a bacterium we previously isolated from *Hydra vulgaris* AEP, which was shown capable of fast resistance formation. Moreover, we are searching for an approach more effective than state-of-the-art phage cocktails (28), which have reportedly been challenged by bacterial resistance (29, 30). To achieve this, we take a deeper look into phage sequential treatments and the factors determining their efficacy and ability to curb bacterial growth.

## RESULTS

### Phage isolation and classification

**Fig 1:**
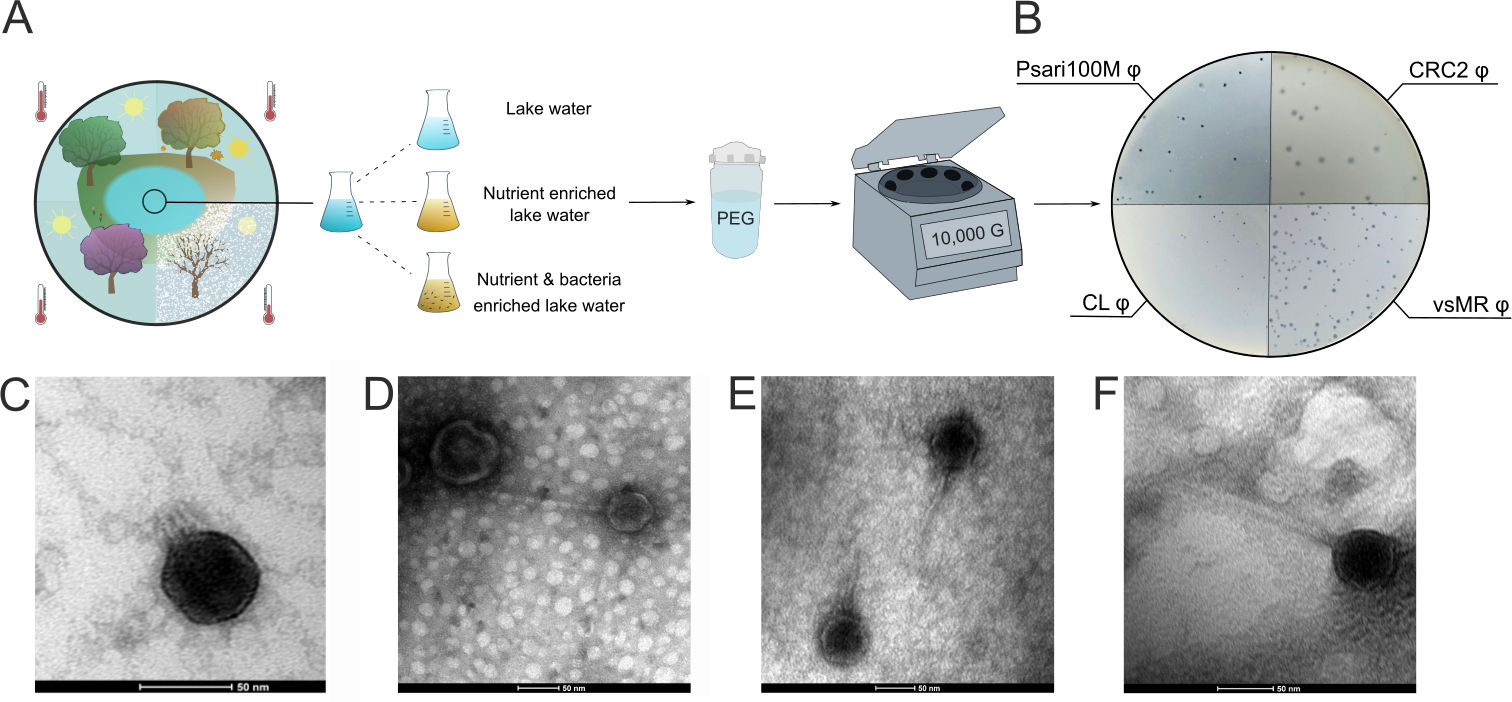
Isolation and classification of *Pseudomonas alcaligenes* phages. (**A**) Scheme to visualize bacteriophage extraction from lake water in February, April, September, and November via nutrient and bacterial enrichment, PEG felling, and centrifugation. (**B**) Image of *Psari100M*, *CL*, *CRC2*, and *vsMR* plaque-forming units in overlay agar. (**C**) Transmission electron microscopy of *Psari100M*. (**D**) Transmission electron microscopy image of *CL* phage. (**E**) Transmission electron microscopy image of *CRC2* phage. (F) Transmission electron microscopy image of *vsMR* phage.

In order to obtain a vast variety of bacteriophages infecting *P. alcaligenes* for our study, we collected lake water (Fig. 1A), and isolated single plaque-forming units (PFUs), resulting in four unique bacteriophages: *Psari100M* φ, *CL* φ*, CRC2* φ, and *vsMR* φ (Fig. 1B).

Psari100M φ formed medium-sized PFU with a diameter of approximately 5 mm and a distinct border. Observation under transmission electron microscopy (TEM) showed that Psari100M had a 20-nm-long tail and a 50-nm-wide icosahedral head. The presence of a small tail indicated that Psari100M belonged to podoviral phages (31, 32). CL φ formed smaller PFU with a distinct border. TEM images of CL set its head at approximately 65 nm width and tail at 150 nm, resulting in a total length of 215 nm. Since the tail was not retractable, it could be classified as a siphoviridal-like phage. CRC2 φ created the largest PFU with a diameter of 10 mm and fuzzy border. The phage had a 50-nm-wide head and a 70-nm-long tail, indicating siphoviridal morphology as well. Finally, the vsMR phage formed medium-sized to small plaques. Its head was 70 nm wide while its tail was 160 nm long. Like our other tailed bacteriophages, its tail was non-contractile, classifying vsMR as a siphoviridal-like phage. All of them belonged to the newly formed class of Caudoviricetes (33) (Fig. 1B through F).

**Fig 2:**
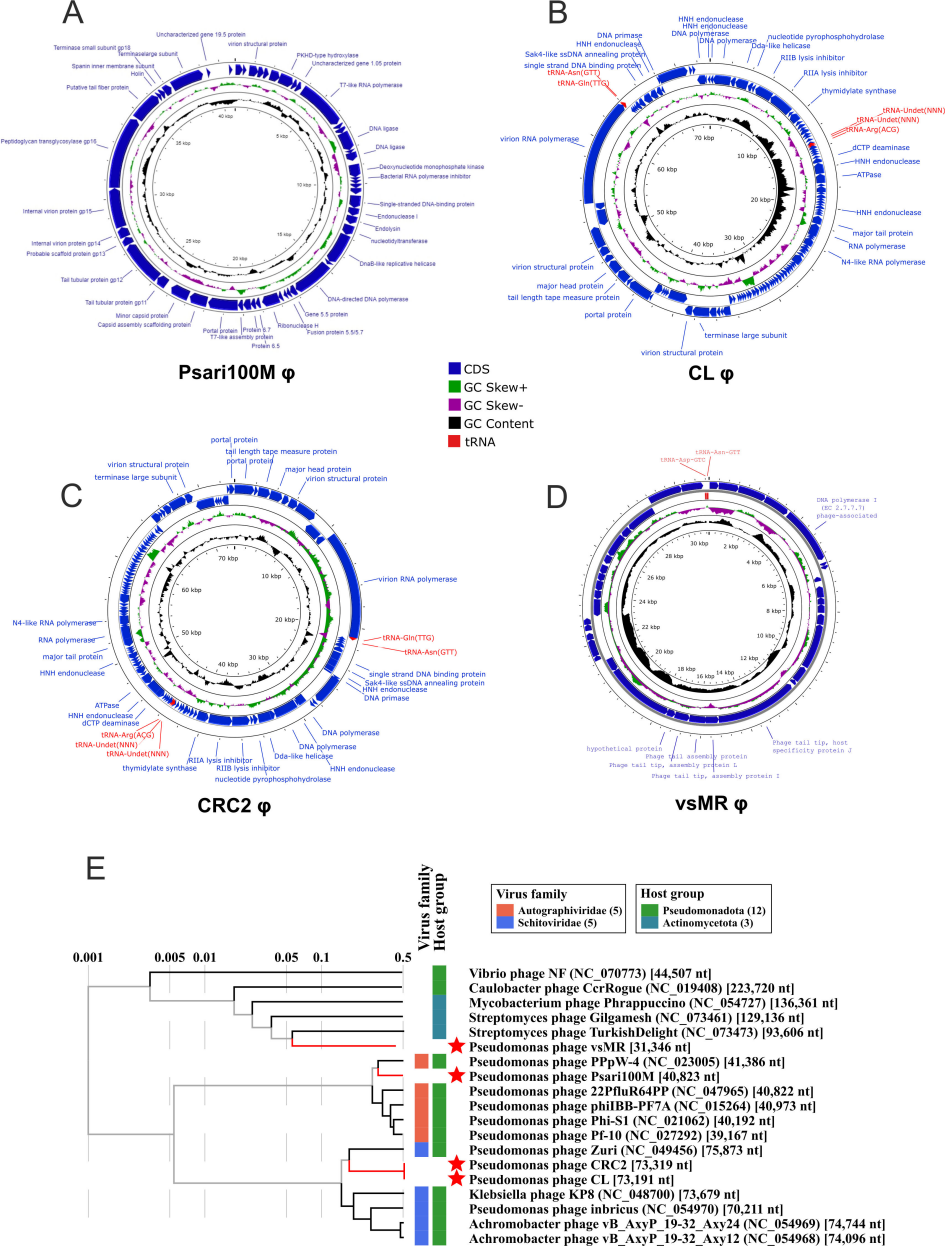
Genome annotation and phylogenetic analysis of *P. alcaligenes* phages, with genomes arranged into circular representations for ease of viewing. (**A**) Annotated genome of Psari100M phage (BankIt: OR687459). (**B**) Annotated genome of *CL* phage (BankIt: OR687461). (**C**) Annotated genome of *CRC2* phage (BankIt: OR687458). (**D**) Annotated genome of *vsMR* phage (BankIt: OR687460). (**E**) Taxonomic tree generated using VipTree to cluster 15 similar phage genomes to Psari100M phage, CRC2 phage, CL phage, and vsMR phage.

Sequencing our bacteriophage genomes showed that the *Psari100M* phage had a genome size of 40,823 bp. While genes for capsid proteins, tail tubular proteins, DNA ligases, and DNA polymerases were found, protein prediction did not reveal an integrase. The *Psari100M* phage was thus unlikely to be a lysogenic phage (Fig. 2A).

The CL phage, at 73,191 bp, contained a major head protein, DNA and RNA polymerases, a helicase, and nucleases, among others. Protein prediction did not identify an integrase within the genome of CL phage, thus indicating CL to be a lytic rather than a lysogenic phage (Fig. 2B).

Analysis of the *CRC2* φ genome showed that it contained a total of 73,319 bp. Similar to the *Psari100M* phage, protein prediction indicated the presence of capsid and tail proteins. Furthermore, it contained RNA polymerases in addition to its DNA toolkit consisting of DNA polymerases, primases and helicases. The *CRC2* φ genome does not code for an integrase, indicating that it may likely be a lytic bacteriophage (Fig. 2C).

The vsMR phage, with a genome containing 31,346 bp, coded for phage tail tip proteins, DNA polymerase, and several tRNAs, according to protein prediction. Though our prediction was not able to identify all encoded proteins, listing some as hypothetical, we did not find any integrases and would therefore hypothesize that vsMR phage is likely lytic in nature (Fig. 2D).

While our bacteriophages presented as double-stranded DNA viruses with high levels of completeness according to predictions, we could not clearly determine whether their genomes were circular or linear. Regardless, we represented their genomes in a circular fashion for ease of view (Fig. 2).

Taxonomic tree analysis based on nucleotides showed that all four phages were neither identical to each other nor to another published phage genome. *Psari100M* was closely related to but not identical to *Pseudomonas* phage *PPpW-4* (NC_023005), while *CL* phage was most similar to a variety of *Pseudomonas*, *Klebsiella*, and *Achromobacter* phages. The closest relative to the CRC2 phage was Pseudomonas phage Zuri (NC_049456). Finally, the vsMR phage showed similarity to Streptomyces phages (Fig. 2E).

### Phage concentration affects bacterial growth

As we have learned previously, the ability of phages to infect a bacterium on solid medium does not guarantee success when infecting its bacterial host in liquid culture (34). Since our goal was to prepare a therapeutic intervention regimen using bacteriophages effective against bacteria in liquid culture and on solid surfaces, we tested infectivity of all our phages in *P. alcaligenes* liquid culture. Additionally, we posed the question of how different phage concentrations would affect the speed and amount of resistance formation. To achieve this, we added dilutions of phage solution with varying titers to *P. alcaligenes* cultured in liquid medium. Rather than merely observing a decline in bacterial growth, we were able to see clear differences in growth patterns based on phage concentration.

**Fig 3:**
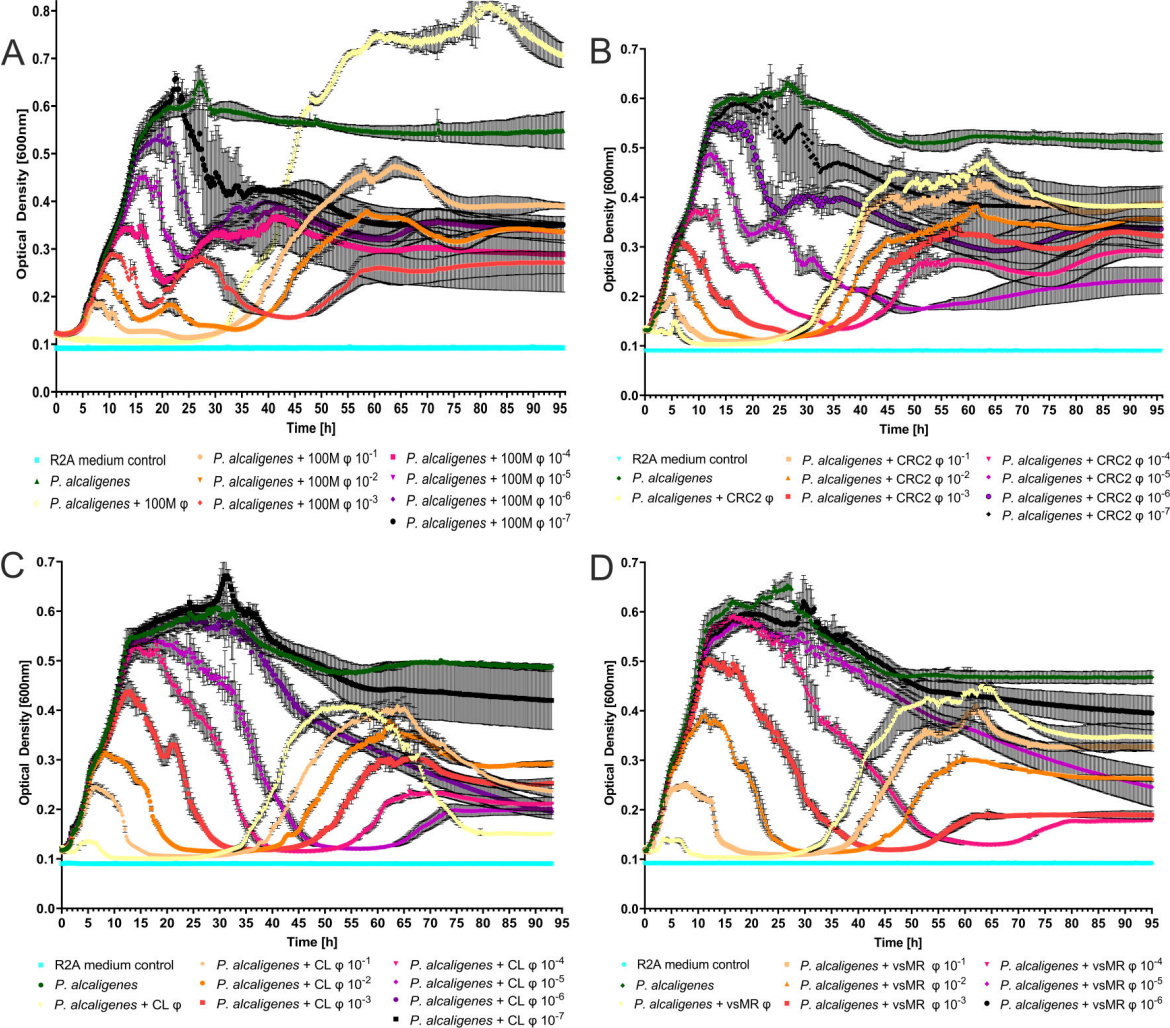
Growth analysis of *P. alcaligenes* infected with different concentrations of each bacteriophage. (**A**) Optical density measured over time to determine bacterial growth of *P. alcaligenes* infected with different concentrations of *Psari100M* phage with standard deviation (*N* = 4). (**B**) OD measured over time to determine growth of *P. alcaligenes* infected with *CRC2* phage dilutions (SD, *N* = 4). (**C**) OD over time of *P. alcaligenes* infected with *CL* phage dilutions (SD, *N* = 4). (**D**) OD over time of *P. alcaligenes* infected with *vsMR* phage dilutions (SD, *N* = 4).

In case of *Psari100M* φ, the highest phage concentration resulted in no initial OD increase until 33 h, after which bacterial growth started and reached an OD 0.8 over the course of 60 h. Adding 5 × 10^6^ PFU/µL resulted in an initial peak at 0.19 OD within 9 h, before growth decreased, and stagnated for 30 h. After 36 h, bacterial growth increased exponentially up to 0.46 OD, before reaching stationary phase. This trend continued in all following dilutions, with higher initial bacterial growth correlating with lower phage concentration. *P. alcaligenes* without phages grew to a maximum OD of 0.65 within 27 h, before reaching tstationary phase. Most cultures with Psari100M φ reached stationary phase at ODs ranging around 0.33 OD, except for the two highest concentrations that stagnated at higher OD and the 49,000 dilution, which stagnated at 0.26 OD (Fig. 3A).

Adding *CRC2* φ to liquid *P. alcaligenes* culture resulted in bacterial growth up to an OD of 0.15 within 5 h. Then, bacterial growth stagnated at 0.1, until it increased exponentially after 30 h to an OD of 0.45. It remained at that level until the 63 h mark, after which OD decreased again. As observed in cultures containing *Psari100M* φ, initial bacterial growth increased inversely proportional to phage concentration, with an initial peak at 0.1 OD in a culture with 1,865,000 PFU, an initial peak at 0.33 OD in a culture with 186,500 PFU, a peak at 0.49 OD in a culture with 1,865 PFU, and so on. Another trend we observed was that secondary bacterial growth began later with decreasing phage concentrations; such that exponential growth started after 30 h in a culture with 186 × 10^6^ PFU, while exponential growth started after 38 h in a culture with 186,500 PFU. This pattern was broken in cultures with less than 2,000 PFU, where the initial peak was followed almost immediately by a secondary peak, before stagnating. Additionally, it could be observed that secondary bacterial growth reached higher OD values when the first peak was lower. All bacterial cultures with CRC2 φ reached stationary growth at ODs ranging from 0.38 to 0.21 (Fig. 3B).

Graphs derived from a dilution series of CL φ phage followed the same trends mentioned above (Fig. 3A and B). As such, lower phage concentrations were again linked to higher initial bacterial growth, followed by later resistance formation and lower secondary growth. Addition of a 10^−7^ dilution, generating low phage concentrations of approximately 4 PFU/µL, resulted in bacterial growth reaching an even higher peak than liquid *P. alcaligenes* culture without phages. Unlike CRC2 and Psari100M, bacterial growth following CL phage infection does not end in stationary phase but rather leads to a decline even after the secondary peak (Fig. 3C).

*VsMR* φ followed the same trend as its predecessors, with initial bacterial growth increasing inversely proportional to a lower initial phage concentration and secondary growth increasing in cultures with higher initial phage concentrations. Notably, final OD values were again stationary and larger than 0, ranging from 0.18 to 0.41. If all bacteria had been eradicated, we would expect a final OD around 0.1, similar to our medium control (Fig. 3D).

When comparing the heights of our first peaks from each phage concentration, we found that they often differed significantly from each other and always showed significant differences compared to our control without phages. Furthermore, we were able to observe again that peak height increased when phages were at lower concentrations (Fig. S2A through D). These results do not however indicate that the highest phage concentration similarly resulted in the lowest overall bacterial load. Instead, our lowest total bacterial loads occurred at MOIs (multiplicities of infection) ranging from one phage per bacterial cell in case of vsMR phage and CRC2 phage to an MOI of 10 in Psari100M phage and MOI of 50 in CL phage (Fig. S2E).

### Bacterial resistance

One could argue that the rise in optical density, from which we inferred bacterial load, was a result of interference by debris from lysed bacteria. To test this, we pelleted *P. alcaligenes* cells from liquid culture and compared the OD of supernatant and pellet, including supernatant derived from liquid bacterial cultures infected with phage. Pelleted bacterial cells displayed an OD of 0.4. Supernatant from culture with and without phages did not display any increase in OD, remaining at a level similar to our medium control at approximately 0.1 OD. Only after 12 h did supernatant from phage-infected bacteria increase in OD (Fig. S1).

Since ODs of both supernatant solutions behaved similarly to our medium control, it does not seem as though cellular debris derived from phage lysis interfered with optical density. Therefore, we hypothesized that bacterial growth was the cause of our observed increase in OD. This would only be possible if *P. alcaligenes* had either become resistant to phages or used a defense mechanism to stop phage infection. Abortive systems and CRISPR Cas systems are among the known defense mechanisms resulting in phage destruction, while resistance mechanisms include the permanent alteration of surface structures to hinder phage attachment or entry of genetic material (35). Analysis of the *P. alcaligenes* genome did not indicate the presence of a CRISPR Cas system, which hints that our phages may not have been destroyed. This was confirmed when we re-isolated phages at the end of our concentration assays (Fig. 3) and used them for spot assays containing unexposed *P. alcaligenes*. These assays developed lytic phage spots, indicating that phages had indeed not been destroyed. Thus, the most likely conclusion was that *P. alcaligenes* had mutated to form resistance without killing phages.

**Fig 4:**
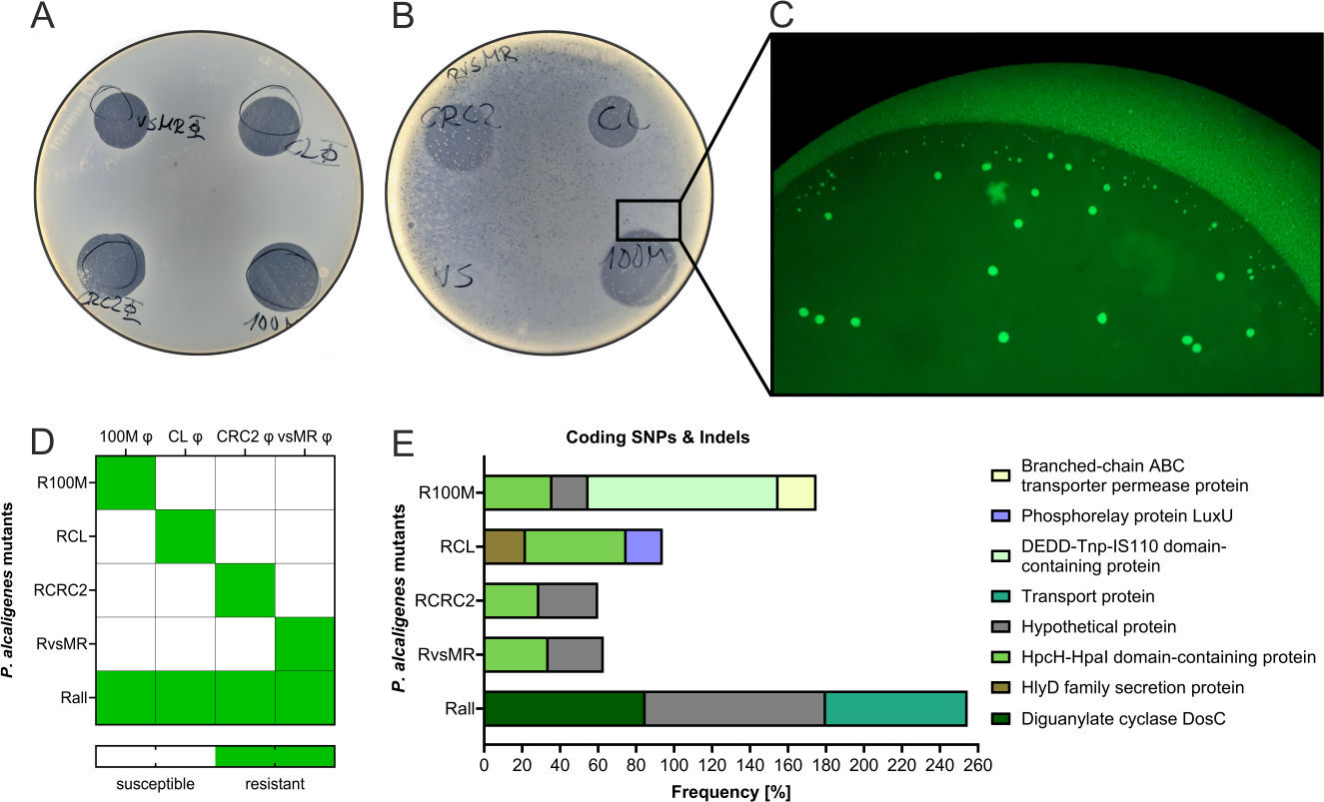
Resistance development in *P. alcaligenes*. (**A**) Spot assay containing *P. alcaligenes* in overlay with *Psari100M*, *CL*, *CRC2*, and *vsMR* phage spots. (**B**) Spot assay, overlay containing *P. alcaligenes* previously infected with and now resistant to *vsMR* phage, while *Psari100M*, *CL*, *CRC2*, and *vsMR* phage were spotted on top. (C) Fluorescent microscopy image of GFP labeled *P. alcaligenes* appearing within spot after 48 h. (**D**) Heatmap depicting which *P. alcaligenes* mutants are resistant to *Psari100M*, *CL*, *CRC2*, or *vsMR* phage. (**E**) Bar chart showing SNPs and Indels within coding regions and frequencies of occurrence within resistant *P. alcaligenes* mutants.

To test for bacterial resistance, we simultaneously re-isolated bacteria at the end of our phage concentration assays (Fig. 3), and investigated their susceptibility to each bacteriophage via spot assays. We compared re-isolated bacteria to negative control *P. alcaligene*s which had not come into contact with phages. Our negative control showed transparent spots where bacterial lysis had occurred, thus reaffirming the lytic ability of our phage solutions (Fig. 4A), while bacteria exposed to phages during phage concentration assays (Fig. 3) did not show lytic spot formation.

Interestingly, we were able to observe the formation of small PFU outside of phage spots, indicating remaining phage activity at the end of the growth assay. Additional spotting of *vsMR* phage on top did not yield a transparent spot. One could hypothesize that the *P. alcaligenes* strain used in this overlay agar became resistant to the initial vsMR φ solution, while the vsMR phages creating these PFUs may have co-evolved to be capable of infecting the resistant *P. alcaligenes* strain (Fig. 4B).

To confirm that our observed lack of infection was not a result of bacterial contamination, we performed spot assays with GFP labeled *P. alcaligenes*. As a result, we observed single colony-forming units (CFUs) within previously transparent phage spots, where CRC2, Psari100M, and CL and vsMR phages had been spotted. Since these CFUs were green fluorescent, we were able to show that these resistant bacteria were indeed *P. alcaligenes*, thus ruling out bacterial contamination (Fig. 4C).

To exclude potential artifacts, we performed another round of spot assays similar to those shown Fig. 4A. Specifically, we exposed *P. alcaligenes* to all four single phages and a phage cocktail in liquid culture rather than re-isolating bacteria from phage concentration assays. After incubation, we observed that resistance formation had occurred at the same time in phage cocktails as it had occurred in mono-phage treatments. As such, bacteria co-cultured with Psari100M did not show a transparent spot where Psari100M was spotted, but showed three lytic spots for CL, vsMR, and CRC2 phages. The same held true for our other phages, resulting in spot assay patterns similar to Fig. 4B. Bacteria co-cultured with the phage cocktail yielded no transparent spots at all, no matter which phage was added. A summary of these observations can be found in Fig. 4D. Furthermore, we named *P. alcaligenes* resistant to all four phages after exposure to cocktails as Rall, while *P. alcaligenes* resistant to Psari100M were named R100M, *P. alcaligenes* to CL phage were named RCL and so on (Fig. 4D).

Genome sequencing of resistant bacteria revealed that *P. alcaligenes* did indeed undergo genomic changes, which allowed us to investigate whether each mutant acquired the same set of mutations or a different pattern of mutations specific to each phage. In R100M, we found four mutations not present in wild-type *P. alcaligenes*. Among them, a mutation within the “DEDD-Tnp-IS110 domain-containing protein” gene occurred with a frequency of 100%, followed by a mutation in HpcH-HpaI domain-containing protein with 36% frequency and a branched-chain ABC transporter permease protein and hypothetical protein at 20%.

Mutants resistant to the CL phage presented the same mutation in the HpcH-HpaI domain-containing protein and two novel mutations in the HlyD family secretion protein and Phosphorelay protein LuxU. *P. alcaligenes* mutants resistant to CRC2 φ and vsMR φ both contained mutations in the previously mentioned hypothetical protein and HpcH-HpaI domain-containing protein with frequencies of approximately 30%. Unlike mutants resistant to one phage, the Rall mutant resistant to our phage cocktail, showed mutations whose frequencies all lie at 100%. Among them is again our hypothetical protein, a mutation in Diguanylate cyclase DosC and one in a transport protein (Fig. 4E).

### Sequential treatment to combat resistance formation

**Fig 5:**
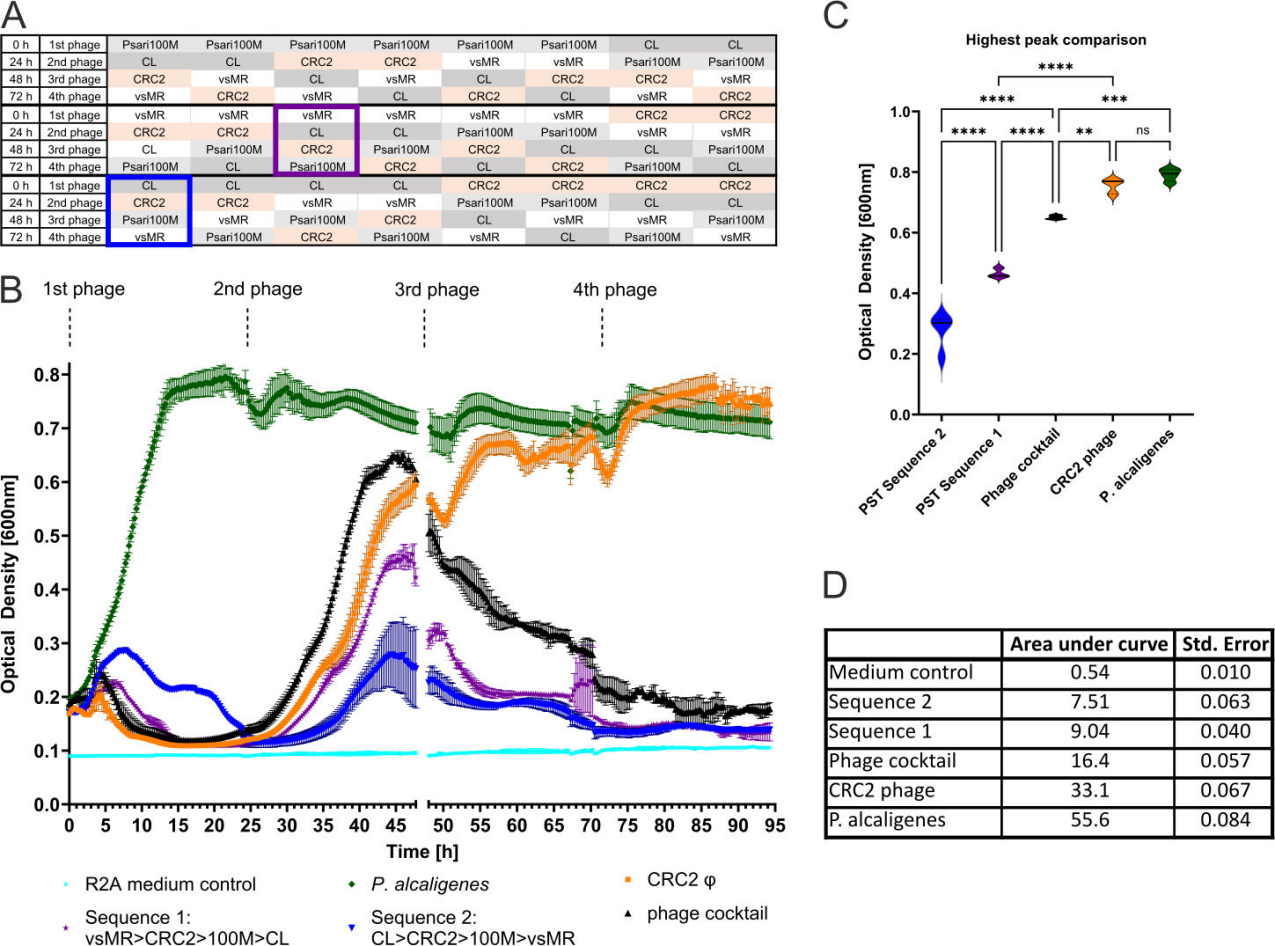
Phage sequential treatment of *P. alcaligenes*. (**A**) Scheme listing all tested phage sequential treatments. (**B**) Optical density measured over time to determine growth of *P. alcaligenes* without phages, *P. alcaligenes* treated with *CRC2* phage, *P. alcaligenes* treated with a phage cocktail and phage sequential treatments Sequence 1 and Sequence 2. Error bars utilize standard deviation (*N* = 4). (**C**) Comparison of highest peaks of each graph found in (B) using Tukey’s test following one-way ANOVA with *F* = 164.2. “****” denotes *P* < 0.0001, "***” denotes *P* = 0.0003, “**” denotes *P* = 0.0073, and “ns” equates to *P* = 0.6179. (D) Area under the curve and its standard error analysis of all graphs visible in (B).

So far, we have shown that, upon exposure to a phage, bacteria became resistant to that one phage while still being susceptible to our other phages (Fig. 4B and D). In addition, administration of a phage cocktail led to resistance formation against all four phages in the same time span it took *P. alcaligenes* to form resistance against single phages. To counteract resistance formation, we considered that phages caused different mutation patterns (Fig. 4E), which led us to hypothesize that addition of one phage after another may force bacteria to adapt to one phage at a time. Such an approach may hypothetically be more efficient than a phage cocktail approach, wherein bacteria could adapt to all phages at once. Thus, we started a new set of growth assays, adding several combinations of our phages to liquid *P. alcaligenes* culture in intervals of 24 h. Due to having four phages, we tested 24 unique PSTs in total. Two notable examples included PST consisting of *vsMR* φ, followed by *CL* φ, followed by *CRC2* φ and then *Psari100M* φ. A second good performer consisted of Sequence 2, starting with *CL* φ, followed by *CRC2* φ, followed by *Psari100M* φ and lastly *vsMR* φ (Fig. 5A).

We tested the efficiency of these phage sequential treatments by comparing each PST to a phage cocktail and monophage treatment, while measuring bacterial growth at OD_600_. The first phage was added to liquid *P. alcaligenes* culture at 0 h and every following phage in intervals of 24 h. Untreated *P. alcaligenes* grew to a maximum OD of 0.8 within 15 h, before entering stationary phase, whereas our phage cocktail reduced bacterial growth, so that its maximum peak occurred at an OD of 0.6 at 45 h. Instead of reaching stationary phase, *P. alcaligenes* growth declined again after 45 h. When PST Sequence 1 was added, bacterial growth began at 28 h and reached a maximum peak of 0.45 OD. PST Sequence 2 resulted in an initial growth peak at 8 h of up to OD 0.28, before decreasing bacterial growth and only reaching a maximum peak of 0.3 at 44 h (Fig. 5B).

Comparing maximum bacterial growth peaks via Tukey’s test showed clearly that the highest peak of PST Sequence 2 was significantly lower than the highest peak observed in all other treatments. Additionally, the highest peak of PST Sequence 1 was significantly lower than peaks caused by our phage cocktail and monophage treatment, thus indicating that phage sequential treatment performed better in terms of avoiding high bacterial density (Fig. 5C).

To gain insight into the impact of phage treatments on the total bacterial load over the course of our experiment, we calculated the area under each curve, finding that PST Sequence 2 performed best at reducing total bacterial growth throughout the entirety of our experiment. Specifically, PST Sequence 2 reduced bacterial growth seven times more compared to untreated *P. alcaligenes* and two times as much compared to our phage cocktail. PST Sequence 1 was the second-best performer, still reducing bacterial growth six times more compared to untreated bacteria and 1.8 times as much compared to the cocktail. Monophage treatment as well as untreated *P. alcaligenes* yielded the highest total bacterial load, though monophage treatment still reduced bacterial load by half compared to untreated bacteria (Fig. 5D).

Spot assays performed after completion of this experimental series indicated that phages were still active and capable of lysing unexposed *P. alcaligenes* cultures. Furthermore, we observed that sequential treatments varied greatly depending on the combination of phages. The results of other tested PST combinations may be found in Fig. S1C and D (*N* = 4).

## DISCUSSION

Over the course of this study, we identified four novel bacteriophages, characterized their infection patterns and tested PST as a promising alternative to phage cocktails in an effort to overcome bacterial resistance. These experiments were conducted using *P. alcaligenes* as a model organism due to its close relatedness to *P. aeruginosa*, one of the leading causes of multi-resistant infections (3). As such, treatments effective in *P. alcaligenes* are potentially applicable to *P. aeruginosa* as well as other “ESKAPE” organisms and may thus be relevant in clinical settings.

In monophage treatment, initial bacterial growth peaks (5–10 h) were likely caused by phage concentrations too low to kill all bacteria at once. As a consequence, consecutive decreases in bacterial growth may have been caused by increasing numbers of replicating phages. This hypothesis is supported by the lack of an initial peak after concentrating Psari100M phages to an MOI of 500 (Fig. 3A). Since initial bacterial growth peaks were at their lowest using high phage concentrations, one could assume that a higher MOI would automatically perform best at curbing bacterial growth. While we did indeed show that a higher MOI produced the lowest initial growth peaks (Fig. S2A through D), this hypothesis does not hold true in terms of total bacterial load. Instead, the lowest total bacterial load was achieved by MOIs between 1 and 10, as we can show by calculating the AUC (area under the curve) of each phage concentration (Fig. S2E).

Secondary growth peaks (~30 h, Fig. 3) on the other hand were the result of resistance formation. This hypothesis was supported by CFU growing within spots after only 48 h (Fig. 4C). Additionally, we observed resistance development to all four phages in our spot assays (Fig. 4D), which was congruent with observations in other studies, where bacteria were capable of forming resistances to several phages at once (36). Especially in cases of very high phage concentrations such as in *Psari100M* (Fig. 3A), secondary bacterial growth may have increased further due to phage concentration methods simultaneously resulting in larger amounts of bacterial debris, which may have served as an additional nutrient source for *P. alcaligenes* (Fig. S1B).

In conclusion, it seems most beneficial to not only reduce initial growth peaks, but to keep bacterial growth curves from fluctuating strongly, which we observed whenever we used an MOI of 1–10. A potential explanation for this may be the arising competition over nutrients between non-resistant bacteria, which made up the first growth peak and resistant bacteria, which account for secondary growth peaks. This concept also applied to our PST, during which we added phages in intervals to keep bacterial growth steadily contained.

This approach seemed to have been successful, since our PST performed better than phage cocktails, as they were more efficient for two reasons; First, maximum growth peaks were reduced the most after PSTs compared to the maximum growth peak we observed after cocktail treatment (Fig. 5B). Comparing both peaks via Tukey’s multiple comparisons test further confirmed that PST outperformed the phage cocktail significantly in terms of subduing bacterial growth (Fig. 5C). Second, we showed that bacterial growth remained stable at a relatively low OD over the course of 95 h in PST, resulting in half as much total bacterial growth, as calculated by AUC, compared to our cocktail treatment (Fig. 5D). Consistently low levels of bacterial growth are especially important in clinical settings, because the immune system has a better chance of dealing with low quantities of bacteria. Additionally, large bursts of bacterial growth followed by lysis as observed in the cocktail treatment can lead to the release of larger amounts of potentially harmful endotoxins (37).

While we highlighted our most successful treatments (Fig. 5B), we observed a high level of diversity in bacterial growth patterns among different phage sequential treatments (Fig. S1C). This was interesting because we added the same phages and concentrations with the only difference being their order of addition. These findings further confirmed observations made by Wright et al. (27), who also concluded that phage order was an important factor in sequential treatments. Our strategy was furthermore substantiated by literature describing similar experiments, alternating between different antibiotics to achieve vulnerability (38), and resistance delay in bacteria (39). While these treatment regimens relied on forcing bacteria to switch between different antibiotic resistance strategies, the same principles applie to phages, assuming our phages use different entry and infection strategies, which mutant analysis seems to imply. As shown in Fig. 4E, where some mutations were shared among resistant *P. alcaligenes* such as the HpCH-Hpal domain protein mutation, others were unique to specific phages such as the DEDD-Tnp-IS10 domain protein mutation only found in bacteria resistant to *Psari100M* phage, as well as the phosphorelay LuxU mutation present in bacteria resistant to *CL* phage only (Fig. 4E).

According to UniProt (40), the DEDD-Tnp-IS10 domain protein found in R100M mutants may potentially contribute to DNA-binding. Therefore, the protein may either be responsible for changing bacterial metabolic activity by acting as a transcription factor, or may directly impact the processing of phage DNA. Meanwhile, the phosphorelay protein LuxU found in RCL mutants, is a phosphorelay sensor with potential implications for bacterial chemotaxis with no previously noted involvement in phage infection. The mutation within HpCH-Hpal domain protein belonging to the aldolase/citrate lyase family, was found to be most similar to a sequence from *Pelagibacterium* and no previously recorded literature regarding its involvement in phage resistance formation either. All in all, more sequencing and potentially proteomic work would be required to further illuminate which mutations among the ones we found play vital roles in the formation of phage resistance.

On the other hand, bacteria resistant to *vsMR* and *CRC2* do not seem to display unique mutations even though bacteria resistant to one phage can still be infected by the other. This could be a result of our focusing on mutations in coding regions, as there were further single nucleotide polymorphisms (SNPs) within non-coding areas, likely because selection pressure is lower in non-coding regions (41). Unfortunately, this also makes it more difficult to pinpoint mutations relevant for resistance formation. Even though a non-negligible part of new SNPs could be found among surface structures which historically have been shown to be common attachment points for phages, such as flagella, channels, and transport proteins (42), mutations do not seem to have high predictive power in our series of experiments.

Another aspect worthy of consideration is the observed ability of phages to increase vulnerability towards antibiotics, as was tested in *Salmonella* by Laure and Ahn (43) as well as Turner et al. (44), who observed an increase in antibiotic sensitivity in *P. aeruginosa* after phage treatment. While we did not experimentally test whether our phages were capable of increasing susceptibility to antibiotics, it may certainly be interesting to follow up on this topic, using our library of phage-resistant mutants.

Thus, we need to look toward different ways to improve phage treatments and to combat resistance formation. Our experiments have opened multiple avenues for future improvements. One important factor with potential predictive power is phage concentrations because of their ability to impact the height of initial and secondary peaks and thus determine the amount and timing of bacterial growth. While literature does not unanimously claim an ideal phage titer for therapy, phage concentrations below 1 × 10^4^ PFU/µL were shown to be less effective *in vitro* in *M. tuberculosis* (22), whereas clinical treatments utilized titers around 1 × 10^8^ (21) or even 1 × 10^10^ (45). While our lowest phage titers range around 5 × 10^8^, and our experimental data clearly depicts phage infectivity, we can be relatively certain that our experiments took place within the infection range of our phages, especially since we saw weak declines in bacterial growth even at incredibly low concentrations such as 100 PFU/µL (Fig. 3).

Further options we have not explored as much include interval times, since we only tested 24 h intervals due to technical limitations. It is possible that different intervals such as 12 h intervals, 30 h intervals, or even mixed intervals may also have an impact on bacterial growth patterns. Nutrients may also influence bacterial growth, though they are more difficult to control for in clinical settings. Another avenue to consider for future experiments is phage receptors, since different bacteriophages use a vast variety of entry points to infect bacteria (46). Recommendations state that phage cocktails that contain phages specifically targeting different entry points of a bacterium may be more efficient (47). This concept would likely also apply to our sequential treatments, such that we could improve upon our combination by specifically searching for bacteriophages targeting different receptors of *P. alcaligenes*. All these factors, along with co-evolution (48), phage diversity (49), and phage engineering to increase infectivity (50, 51), could all build towards improved phage treatments.

When looking at resistant bacterial mutants, their fitness often determines whether a mutation can be exploited for treatment purposes, since gain of bacterial resistance can be part of a trade-off that results in a loss of function alongside resistance formation. Evidence of such tradeoffs has been found in antibiotic-resistant *E. coli* which traded resistance for a reduction in growth rates (52), as well as in phage-resistant bacteria (53). Additionally, it could be interesting to perform follow-up experiments when working with host-associated organisms, to look for trade-offs that reduce fitness in terms of host-interactions and colonization capacity.

For future experiments, it might be particularly beneficial to include experiments that select for phages targeting different entry points, to make bacterial resistance formation more challenging while also utilizing improved administration intervals and concentrations and to specifically select for phages that generate mutants with lower fitness and thus lower chances of survival and persistence. All of these factors may improve the success of a phage treatment in clinical settings.

## MATERIALS AND METHODS

### Phage collection

Lake water was collected in February, April, September, and November, to collect a number of diverse phages. Samples were warmed up to room temperature (RT) overnight and divided into three 500-mL flasks. One sample was enriched with R2A broth (Neogen), one was enriched with R2A and *Pseudomonas solani* T3 (genome accessible in the GenBank database under the accession number CP158373), and one was not modified. Water samples were incubated at RT and 150 rpm overnight, before they were filtered (grade 595 1/2, Whatman). Filtrate was transferred to 250 mL centrifuge bottles (Beckman Coulter, Polycarbonate) and centrifuged at 10,000 rpm for 30 min using an Avanti JXN centrifuge (Beckman Coulter, JA-14 fixed angle rotor) to pellet bacteria. Supernatant was filtered using 0.2 µm filters (Whatman, Sigma-Aldrich) to remove the remaining bacteria. We dissolved 10% (wt/vol) polyethylene glycol (Sigma-Aldrich) in the supernatant solutions, while they rested on ice for 2 h and centrifuged all samples at 10,000 rpm for 30 min. Pellets were re-suspended using 3 mL SM buffer (50 mM Tris–HCl, 100 mM NaCl, 8 mM MgSO_4_ at pH 7.5). Phage solution was stored at 4°C with 10% (vol/vol) chloroform.

### Spot assays

Overlay agar was prepared by dissolving 1.2 g Neogen R2A broth and 1.6 g agarose in 400 mL sterile H_2_O. The solution was autoclaved at 121°C and stored at 50°C. 1 mL of *P. alcaligenes* culture at 1.3 MF-U was added to 4 mL of overlay, distributed on top of an R2A agar plate and left to cool. About 10 µL of phage solution per spot was added on top. Plates were incubated at RT for 24 or 48 h, depending on temperature.

### Phage propagation

Spot assays were prepared using phage solution after collection. Spots were cut out and placed into 2 mL liquid *P. alcaligenes* culture and incubated at 18°C for 12 h. The phage-bacteria mixture was filtered using 0.2 µm pore filters to remove bacteria and the resulting phage solution added to 10 mL bacterial culture to incubate overnight. This step was repeated with 50 mL of *P. alcaligenes* culture to obtain highly concentrated phage solution. 10% (vol/vol) chloroform was added for conservation.

### Phage isolation

Purity of phages was achieved by preparing dilution series of phage mixtures and isolating single PFU during plaque assays. Dilution series were prepared by diluting phage mixtures in R2A, after which 10 µL from each dilution step were added to 4 mL overlay agar and 1 mL *P. alcaligenes*, to prepare a plaque assay. Overlay was plated and the plaque assay was incubated at RT for 30 h. Single plaque-forming units were excised from each plate and amplified in liquid bacterial culture until we reached a total volume of 500 mL. Bacteria were removed via centrifugation at 10,000 rpm (Beckman Coulter, JA-14 fixed angle rotor) and the resulting phage solution, which was derived from a single PFU and thus purified, was split. One half was used for DNA extraction (see below) while the other half was subjected to PEG precipitation and centrifuged at 10,000 rpm. The phage pellet was re-suspended in 50 mL SM buffer and conserved using 10% (vol/vol) chloroform and stored in the fridge at 4°C. Purity was further controlled by transmission electron microscopy and genome sequencing of DNA isolated from the same purified stocks. All phage stocks were confirmed as pure as no other genomes or multiple genotypes were co-assembled (see below).

### Transmission electron microscopy

About 5 µL of isolated phage solution was collected for morphological characterization via negative staining. Samples were stained with 0.5% (wt/vol) aqueous uranyl acetate (54) and visualized using a FEI Tecnai G2 Spirit BioTWIN transmission electron microscope at 80 kV with a magnification of 40,000–100,000×.

### Phage genome extraction

DNA of *Psari100M* and *CL* phage was extracted using CTAB extraction buffer (100 mM Tris at a pH of 8, 3 M NaCl, 20 mM EDTA, and 3% cetyltrimethylammonium bromide), as described in our previous report (34). DNA was re-suspended in 40 µL DNase-free water and stored at −80°C before sequencing. CRC2 and vsMR DNA were extracted using the DNeasy Blood & Tissue Kit (Qiagen).

### Phage genome sequencing and assembly

The genomic DNA was sequenced with the MinION nanopore technology (Oxford Nanopore Technologies, Oxford, UK) using a MinION Flongle Flow Cell (Cat. No. FLO-FLG001) with the Flow Cell Priming Kit (Cat. No. EXP-FLP002) and the Rapid Sequencing Kit (Cat. No. SQK-RAD004), following the manufacturer’s protocols. The super-accurate model of Guppy (Oxford Nanopore Technologies plc., Version 5.0.11 + 2b6dbff, dna_r9.4.1_450bps_sup) was used for basecalling. Raw reads were adapter trimmed with Porechop v0.2.4 (55) and assembled with Canu v2.2 (56), and the contigs were polished twice with Medaka v1.4.3 with model r941_min_sup_g507 (57). Assembly quality and completeness were assessed with CheckV v1.0.1 (58) and manual inspection.

### Phage genome annotation

Open reading frame (ORF) prediction and functional annotation of phages were performed using a combination of PHANOTATE (59) Pharokka (60), Prodigal (61), Prokka (62), Bakta (63), GeneMarkS (64) RAST (65), and Balrog (66). Consensus gene calls and best hit predicted protein similarity searches were made using PHROGs (67), eggNOG (68), PFAM (69), PhaLP (70), and ACLAME (71). Databases were curated manually. Putative transfer RNA (tRNA) genes were identified using ARAGORN (72) and tRNAScan-SE (73). The graphical genome map was generated with the CGView server tool (74) and grouped by PHROGs functional categories. The classification into head, neck, and tail proteins of tailed bacteriophages was done with VIRFAM (75).

### Phylogenetic tree analysis

*Pseudomonas* phages were classified with other reference phages based on genome-wide similarities using VipTree v4.0 (76). A proteomic tree was generated by BIONJ based on a genomic distance matrix, mid-point rooted, and a tree was regenerated by selecting the closest 15 reference phages according to their highest genomic similarity (S-G) scores. Branch lengths were log-scaled from the root, lengths are based on the genomic similarity score S-G values (normalized tBLASTx scores).

### Phage solution preparation for concentration assays

Isolated phage solution (see “Phage isolation” above) was added to *P. alcaligenes* in liquid culture and incubated overnight. Bacteria were removed via centrifugation at 4,600 rpm (SORVALL Heraeus fixed angle rotor 75,006,445), filtration through 0.2 µm membrane and addition of 10% (wt/vol) chlorofom, to re-obtain phage solution. These steps were repeated to increase the concentration of each of our phage solutions. Psari100M was concentrated to a maximum of 12.5 × 10^9^ PFU/µL, CRC2 phage was concentrated to a maximum of 186 × 10^6^ PFU/µL, vsMR phage was concentrated to a maximum of 1.35 × 10^9^ PFU/µL, and CL phage to a maximum of 41 × 10^6^ PFU/µL. Phage solution was diluted with R2A medium to prepare a dilution series of each phage solution.

### Ninety-six-well plate growth assays

Bacterial growth was analyzed via Optical Density measurements at 600 nm using a Spark TECAN plate reader and 96-well plates (CELLSTAR, Greiner bio-one). All experiments were performed with a total of four wells serving as replicates for each treatment (*N* = 4). Sterile R2A medium and S medium served as negative controls, respectively. *P. alcaligenes* cultures were grown overnight and diluted to an OD_600_ of 0.12 before 200 µL of bacterial culture was added to wells. An OD_600_ of 0.12 contained approximately 10^7^ CFU/mL. Phage solution (20 µL) was mixed in when specified. The plate reader was set to 18°C, low humidity, and 150 rpm. Measurements were taken in 15 min intervals. In the end, 100 µL of bacterial culture was used for spot assays.

### Phage solution preparation for cocktail and PST

Phage mixture for cocktails was prepared according to the amplification step (see above) and then mixed in a 1:1:1:1 ratio, adding 1 mL of each phage solution. The titers of each phage as obtained via PFU counts consisted of 5 × 10^9^ PFU/µL for 100M phage, 577 × 10^6^ PFU/µL for CL phage, 6.7 × 10^9^ PFU/µL for CRC2 phage, and 1.9 × 10^9^ PFU/µL for vsMR phage. Titers apply to PST experiments as well (Fig. S1C; Fig. 5B). These phage concentrations result in an MOI of 500 for Psari100M phage, an MOI of 58 for CL phage, 670 for CRC2 phage, and an MOI of 190 for vsMR phage.

### Mutant picking

About 100 µL of *P. alcaligenes* solution was extracted after growth assays, distributed onto R2A agar, and left to incubate for 24 h. Colonies were picked, grown in liquid culture, and their retained resistance confirmed via spot assays. 2 mL was used for DNA extraction.

### Bacterial DNA extraction

*P. alcaligenes* DNA was extracted using the DNeasy Blood & Tissue Kit (Qiagen).

### Mutant sequencing and SNP calling

*P. alcaligenes* mutants were sequenced by Eurofins using a standard genomic library and an Illumina NovaSeq set to NovaSeq 6000 S4 PE150 XP mode. SNPs were identified via Variant Analysis Pipeline v2.6.8, consisting of raw sequence data analysis (77), mapping (78) alignment and SNP calling using Sentieon’s HaplotypeCaller (79). During this analysis, we compared all our mutants to our “wild-type” *P. alcaligenes* T3 strain, which we isolated from lab-cultured *Hydra* which has thus not been previously exposed to our bacteriophages.

### Statistical analysis

Bacterial growth assays performed at OD_600_ were analyzed using GraphPad Prism Version 10.2.0 for Windows. Peaks were identified and then compared based on their height using one-way ANOVA followed by Tukey’s multiple comparisons test. Entire graphs were compared by utilizing an area under the curve calculation with an OD of 0.1 set as the baseline.

## Data Availability

Assembled phage genomes can be viewed at GenBank under accession numbers OR687458, OR687459, OR687460, and OR687461. Raw sequences of our *P. alcaligenes* mutants can be found at the SRA using accession code PRJNA1068988. The *P. solani* T3 genome is accessible in the GenBank database under the accession number CP158373.

## SUPPLEMENTAL MATERIAL

Figure S1 msphere.00707-23-s0001.svg All PST treatments and growth controls.

Figure S2 msphere.00707-23-s0002.svg Statistical analyses of phage concentration analysis.

ASM does not own the copyrights to Supplemental Material that may be linked to, or accessed through, an article. The authors have granted ASM a non-exclusive, world-wide license to publish the Supplemental Material files. Please contact the corresponding author directly for reuse.
